# Development and Validation of a Novel Metabolic-Related Signature Predicting Overall Survival for Pancreatic Cancer

**DOI:** 10.3389/fgene.2021.561254

**Published:** 2021-05-28

**Authors:** Junyu Huo, Liqun Wu, Yunjin Zang

**Affiliations:** Liver Disease Center, The Affiliated Hospital of Qingdao University, Qingdao, China

**Keywords:** pancreatic cancer, metabolic, prognostic, signature, The Cancer Genome Atlas

## Abstract

Recently, growing evidence has revealed the significant effect of reprogrammed metabolism on pancreatic cancer in relation to carcinogenesis, progression, and treatment. However, the prognostic value of metabolism-related genes in pancreatic cancer has not been fully revealed. We identified 379 differentially expressed metabolic-related genes (DEMRGs) by comparing 178 pancreatic cancer tissues with 171 normal pancreatic tissues in The Cancer Genome Atlas (TCGA) and the Genotype-Tissue Expression project (GTEx) databases. Then, we used univariate Cox regression analysis together with Lasso regression for constructing a prognostic model consisting of 15 metabolic genes. The unified risk score formula and cutoff value were taken into account to divide patients into two groups: high risk and low risk, with the former exhibiting a worse prognosis compared with the latter. The external validation results of the International Cancer Genome Consortium (IGCC) cohort and the Gene Expression Omnibus (GEO) cohort further confirm the effectiveness of this prognostic model. As shown in the receiver operating characteristic (ROC) curve, the area under curve (AUC) values of the risk score for overall survival (OS), disease-specific survival (DSS), and progression-free survival (PFS) were 0.871, 0.885, and 0.886, respectively. Based on the Gene Set Enrichment Analysis (GSEA), the 15-gene signature can affect some important biological processes and pathways of pancreatic cancer. In addition, the prognostic model was significantly correlated with the tumor immune microenvironment (immune cell infiltration, and immune checkpoint expression, etc.) and clinicopathological features (pathological stage, lymph node, and metastasis, etc.). We also built a nomogram based on three independent prognostic predictors (including individual neoplasm status, lymph node metastasis, and risk score) for the prediction of 1-, 3-, and 5-year OS of pancreatic cancer, which may help to further improve the treatment strategy of pancreatic cancer.

## Introduction

Despite the great progress made in treating pancreatic cancer over the last few decades, the prognosis has not been effectively improved (Neoptolemos et al., 2018). Genetic concepts and tools are increasingly being applied to clinical practice, especially in precision medicine (Lomberk et al., 2019). However, the biomarkers related to the prognosis of pancreatic cancer are still limited.

Recently, more and more evidence has revealed the significant effect of the reprogrammed metabolism on pancreatic cancer in terms of carcinogenesis, progression, treatment, and prognosis (Qin et al., 2020). The so-called metabolic reprogramming refers to the significant changes in metabolic patterns during cell carcinogenesis, which involves glycolysis, tricarboxylic acid cycle, oxidative phosphorylation, as well as metabolism of amino acid, fatty acid, and nucleic acid (Ward and Thompson, 2012; Huo et al., 2021a,b). During proliferation, tumor cells rely on metabolic reprogramming to provide nutrition, energy, and biosynthetic activity (Pavlova and Thompson, 2016; Neoptolemos et al., 2018). Pancreatic cancer is a malignant tumor with metabolic heterogeneity. Changes in glucose, lipid metabolism as well as amino acid in pancreatic tumors, from cells to microenvironment, and even at the systemic level, can significantly impact tumor progression (Daemen et al., 2015; Qin et al., 2020). Even for the same patients with pancreatic cancer, the metabolic gene expression of the primary focus and the metastatic focus were relatively different (Chaika et al., 2012; Qin et al., 2020). Although the metabolic targeted therapy for pancreatic cancer is not mature at present (Biancur and Kimmelman, 2018), successive clinical trials have shown that metabolic treatment of pancreatic cancer may improve the prognosis of patients (Zachar et al., 2011; Raez et al., 2013; Alistar et al., 2017). Hence, more metabolic biomarkers related to pancreatic cancer prognosis need to be identified. Considering that the effective clinical treatment of pancreatic cancer is still limited, it is urgent to explore new treatment strategies.

The microenvironment around pancreatic cancer cells is composed of immune cells, stellate cells/fibroblasts, and extracellular matrix (ECM). The rapid proliferation of tumor cells leads to a lack of nutrients in the microenvironment, increased release of lactic acid and other metabolites, and metabolic remodeling such as hypoxia and oxidative stress imbalance. Pancreatic cancer cells rely on metabolic reprogramming to adapt to the lack of energy and nutrition in the tumor microenvironment, abnormal oxidative stress, and so on (Bapat et al., 2011). Therefore, it is necessary to deeply understand the impact of metabolic reprogramming on the occurrence and development of pancreatic cancer, so as to provide new ideas for the targeted intervention of metabolic characteristics for the treatment of pancreatic cancer.

In this study, we identified metabolic genes with different expressions between pancreatic cancer and normal tissues through the TCGA and GTEx databases and explored their prognostic value. The prognostic model, composed of 15 metabolic genes, can accurately predict the survival rate of pancreatic cancer and is an independent predictor related to prognosis. In addition, we integrate the GEO database and ICGC database to verify the model and build a survival predictive nomogram.

## Materials and Methods

### Data Collection

**FIGURE 1:**
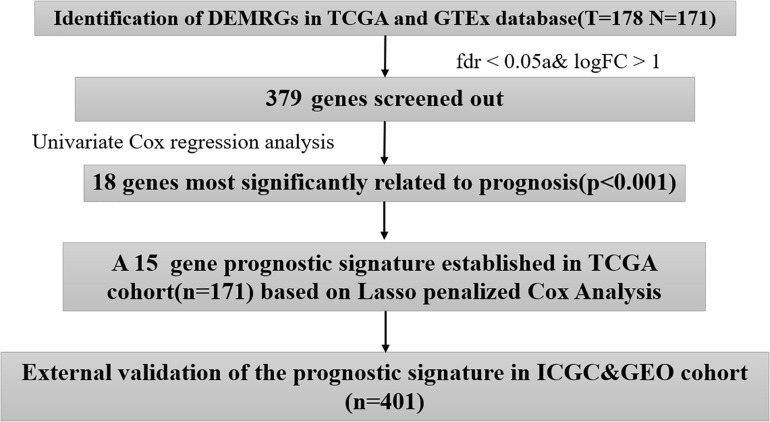
Work flowchart.

We obtained the mRNA sequencing data from The Cancer Genome Atlas (TCGA)^1^ as well as the Genotype-Tissue Expression project (GTEx) (including 178 cancer samples and 171 normal samples). Corresponding clinical data (including the age, gender, survival time, survival status, histological grade, AJCC–TNM stage, presence of new tumors after initial treatment, number of lymph node metastasis, and individual tumor status) were downloaded from UCSC Xena^2^. The mRNA sequencing data together with the corresponding clinical data were downloaded from the International Cancer Genome Consortium (ICGC) (including PACA-AU and PACA-CA, *n* = 273)^3^ and the Gene Expression Omnibus (GEO) (including GSE62452 and GSE57495, *n* = 128)^4^. The work flow chart is shown in Figure 1. R package “sva” was employed to remove batch effects between different datasets; the “sva” package supports surrogate variable estimation with the “sva” function, direct adjustment for known batch effects with the “ComBat” function, and adjustment for batch and latent variables in prediction problems with the “fsva” function (Leek et al., 2012). The study excluded patients whose survival time was less than 1 month and included a total of 572 patients with pancreatic cancer. The acquisition of the above data follows the regulations and permissions of the corresponding database, and does not need to be approved by the local ethics committee.

### Identification of Differentially Expressed Metabolic-Related Genes

We extracted 2,752 metabolism-related genes from mRNA sequencing data of TCGA and GTEx database, which encoded all known human metabolic enzymes and transporters (Possemato et al., 2011). Differential expression of metabolic genes was identified by R package “limma”; false discovery rate (FDR) < 0.05 and log fold change (FC) absolute value >1 were set as the criteria. We also used R package “clusterProfiler” to annotate the gene ontology (GO) and the Kyoto Encyclopedia of Genes and Genomes (KEGG) functions of DEMRGs. The items were recognized with a *p*-value threshold less than 0.05.

### Identification of Prognostic-Associated Metabolic Genes

We used univariate Cox regression analysis to identify DEMRGs related to prognosis. *p* < 0.001 was considered to have a significant effect on prognosis.

### Construction of Prognostic Model in the Cancer Genome Atlas Cohort

One hundred seventy-one samples with completed prognostic information in the TCGA cohort were used for prognostic model construction. We used Lasso regression to narrow the range of prognostic genes, remove overfitting between genes, and calculated risk scores according to Lasso regression coefficients. The risk score is equal to the sum of Lasso regression coefficient of each mRNA multiplied by the normalized expression levels of each mRNA. The median risk score was taken into account to divide patients into two groups: high risk and low risk. Lasso regression analysis was carried out by using R-package “glmnet”; Kaplan–Meier (KM) survival curve was drawn with the R-package “survminer.” Log-rank test evaluated if the survival curve was different, a *p*-value of less than 0.05 was considered to be statistically significant, using R-package “survivalROC” to access the accuracy of risk score. A higher AUC (area under the ROC curve) value generally represents a higher prediction accuracy.

### Assess Whether the Risk Score Could Predict Prognosis Independently

We used univariate and multivariate Cox regression analysis for determining if the risk score was an independent predictor of the prognosis of pancreatic cancer. *p* < 0.05 was considered with statistical significance.

### Analysis of the Association Between the Risk Score and the Clinical Characteristics

We used Wilcoxon signed-rank test (two groups) or Kruskal–Wallis (≥ two groups) for analyzing how risk score affected the clinicopathology. *p* < 0.05 was considered with statistical significance. Boxplot was generated using the “beeswarm” package in the R software.

### External Validation of the Prognostic Model in International Cancer Genome Consortium and Gene Expression Omnibus Cohort

For testing the universality exhibited by the risk score, we integrated 401 pancreatic cancer patients from the ICGC database and GEO database as an external testing cohort. The risk score exhibited by each patient was calculated following the formula and was classified according to the uniform risk group cutoff value. The R package “survminer” was used to generate the Kaplan–Meier survival curve between the two groups, and log-rank assisted in confirming if the survival curve was significantly different (Huo et al., 2020).

### Gene Set Enrichment Analysis Between Different Risk Groups

We conducted GSEA in the populations of the two groups, exploring the potential mechanism of prognostic models affecting prognosis, selecting an annotated gene set file (c2.cp.v7.1.symbols.gmt) as the reference gene set. We set the threshold at nom *p*-value < 0.05.

### Analysis of the Association Between the Risk Score and the Tumor Immune Cell Infiltration

We used TIMER [TumorImmune Estimation Resource, which provided the levels of six tumor-infiltrating immune cells in 10,897 cancer samples (32 types of cancer) from the TCGA database] and CIBERSORT algorithms (using microarray data and a predefined immune signal matrix, estimated the proportion of 22 tumor-infiltrating immune cells in a given sample) to quantify the proportion of immune cell infiltration in tumor tissue (Li et al., 2017; Chen et al., 2018).

### Building a Survival Predictive Nomogram

We incorporated independent prognostic factors into a nomogram to construct a combined model for predicting the OS of pancreatic cancer. The advantage of a nomogram is that each patient can get his or her own specific total score and find the corresponding survival rate on the nomogram, which makes the prognosis assessment more personalized, and we also used calibration curve, concordance index, and ROC curve for verifying the precision exhibited by the combined model. The abscissa of the calibration chart is the predicted survival rate, and the ordinate is the actual survival rate. The closer the predicted survival rate is to the actual survival rate, the higher the overlap between the calibration curve and the reference line. The nomogram was built with R package “rms”.

## Results

### Function Annotation of Differentially Expressed Metabolic-Related Genes

**FIGURE 2:**
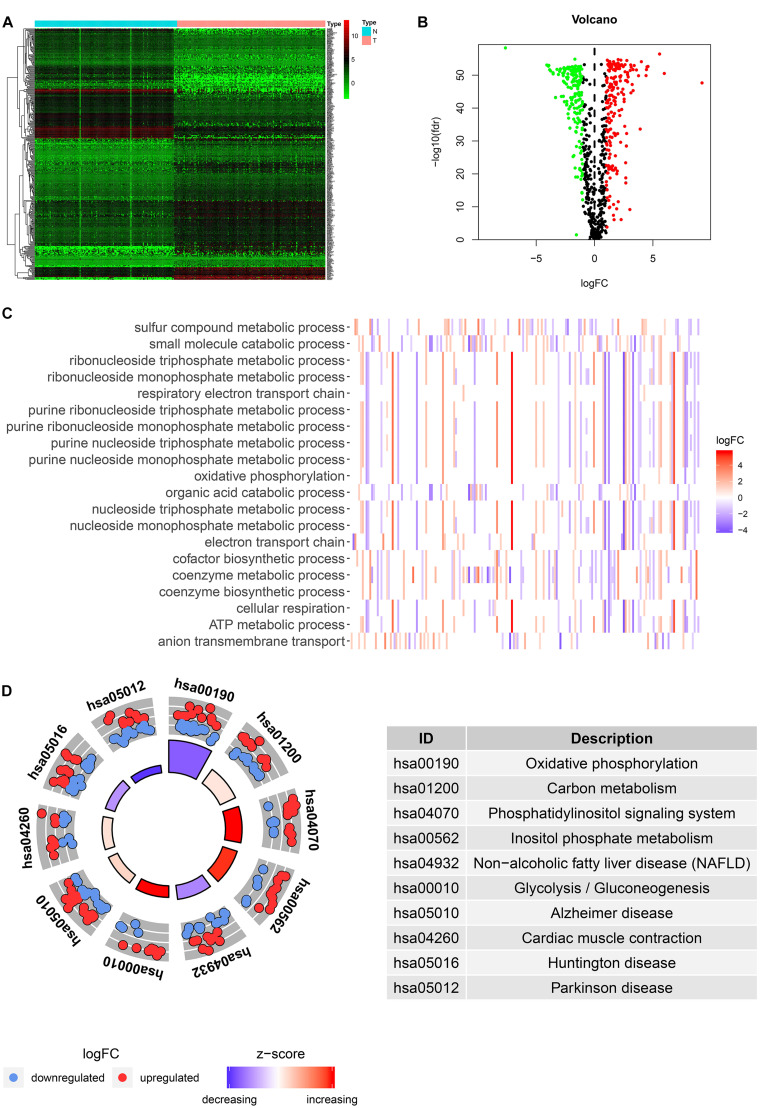
Identification and functional enrichment analysis of differentially expressed metabolic-related genes (DEMRGs). **(A)** Heat map, volcano map, and boxplot of DMRGs. **(B)** Gene Ontology (GO) enrichment analysis of DMRGs. **(C,D)** Kyoto Encyclopedia of Genes and Genomes (KEGG) enrichment analysis of DMRGs.

Among the 379 differential genes, there were 169 and 210 upregulated genes in normal tissues and tumor tissues, respectively (Figures 2A,B). They are mainly involved in a variety of metabolic processes, such as small molecular catabolism, coenzyme metabolism, carbon metabolism, oxidative phosphorylation, and so on (Figures 2C,D).

### Identification of Prognostic Differentially Expressed Metabolic-Related Genes

**FIGURE 3:**
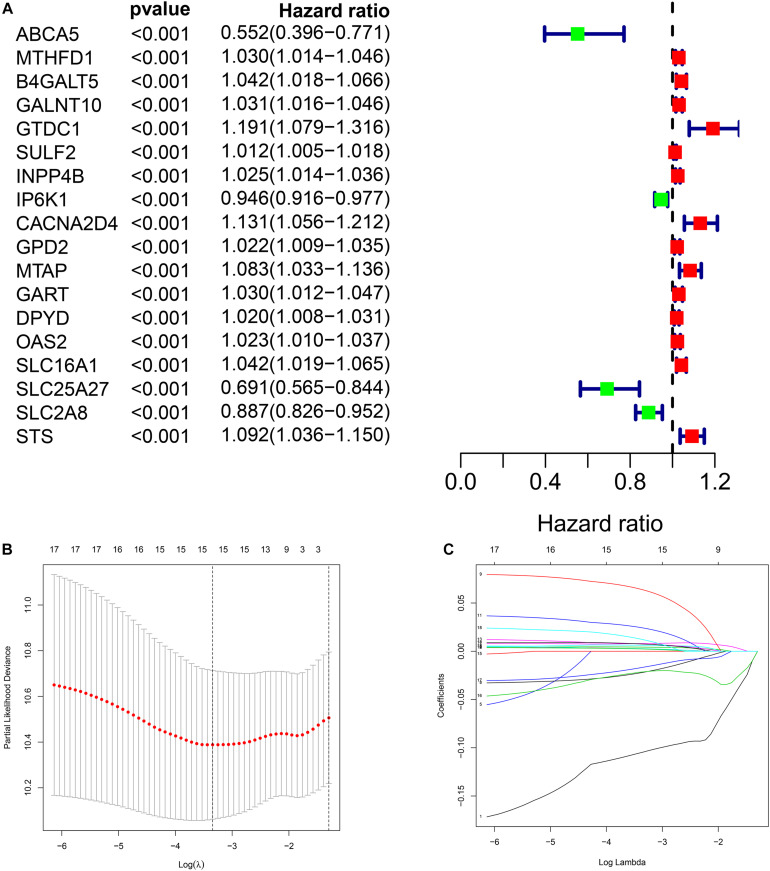
Construction of metabolic prognostic model in The Cancer Genome Atlas (TCGA) cohort. **(A)** Forest plot of prognostic DMRGs. **(B,C)** Lasso regression analysis.

Through univariate Cox regression analysis, we screened 18 genes most significantly related to prognosis (*p* < 0.001) from the 379 DEMRGs, of which four genes were protective factors of prognosis and 14 genes were risk factors (Figure 3A).

### Prognostic Model Construction in the Cancer Genome Atlas Cohort

**FIGURE 4:**
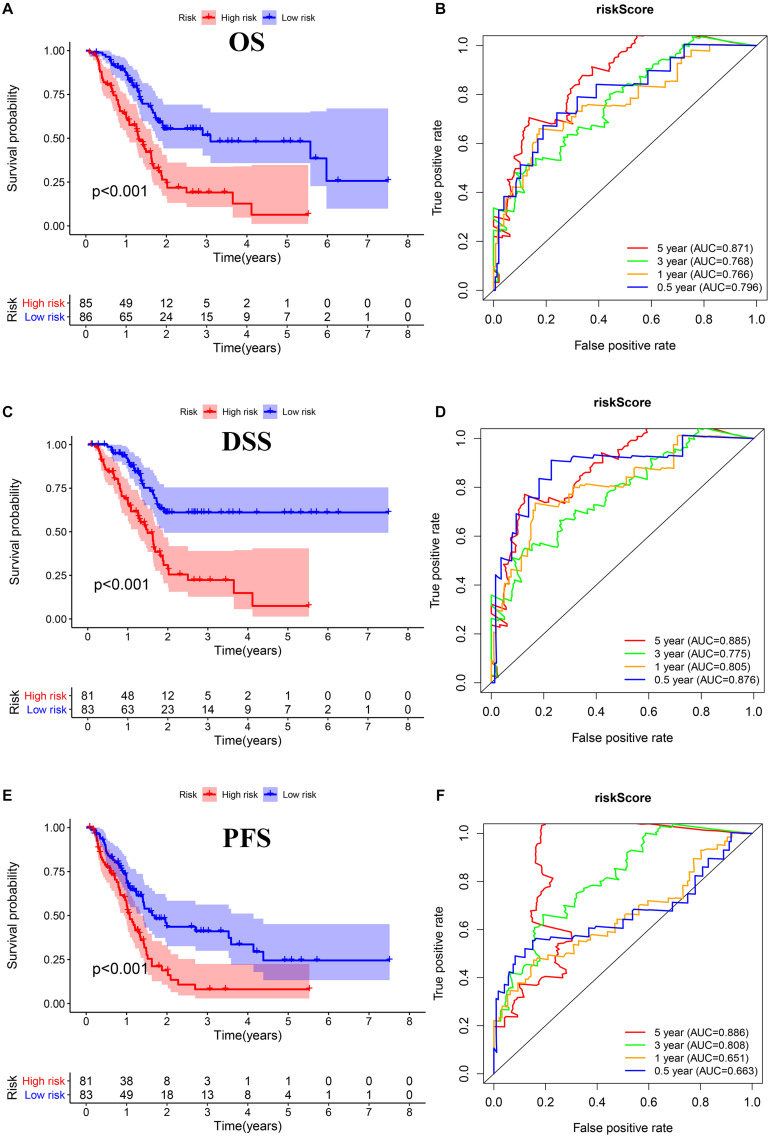
Survival assessment of the prognostic model in TCGA cohort. **(A,B)** Kaplan–Meier survival analysis and time-dependent receiver operating characteristic (ROC) analysis of overall survival (OS). **(C,D)** Kaplan–Meier survival analysis and time-dependent ROC analysis of disease special survival (DSS). **(E,F)** Kaplan–Meier survival analysis and time-dependent ROC analysis of progression-free survival (PFS).

We performed Lasso regression analysis on the above prognostic genes, and after 1,000 cross-validations, the error of a prognostic model containing 15 genes is the smallest (Figures 3B,C). The risk score is equal to the sum of Lasso regression coefficient of each mRNA multiplied by the normalized expression levels of each mRNA. Table 1 lists the calculation coefficient of the risk score. The median risk score (0.655) was taken into account to divide patients into two groups. The group with a high risk exhibited a significantly lower overall survival rate (OS), disease-specific survival rate (DSS), and progression-free survival rate (PFS) compared with the group with a low risk (Figures 4A,C,E). The AUC for 1-year OS was 0.766, for 3-year OS, 0.768, and for 5-year OS, 0.871 (Figure 4B); The AUC for 1-year DSS was 0.805, for 3-year DSS, 0.775, and for 5-year DSS, 0.885 (Figure 4D); The AUC for 1-year PFS was 0.651, for 3-year PFS, 0.808, and for 5-year PFS, 0.886 (Figure 4F). The risk score distribution is shown in Supplementary Material 1. Accordingly, the risk score can be reliably applied for predicting pancreatic cancer patients’ prognosis.

**TABLE 1:** Model gene list and coefficient.

Gene symbol	Gene name	Coef
ABCA5	ATP Binding Cassette Subfamily A Member 5	−0.09947
MTHFD1	Methylenetetrahydrofolate Dehydrogenase, Cyclohydrolase And Formyltetrahydrofolate Synthetase 1	0.001956
GALNT10	Polypeptide N-Acetylgalactosaminyltransferase 10	0.004045
SULF2	Sulfatase 2	0.002094
INPP4B	Inositol Polyphosphate-4-Phosphatase Type II B	0.008312
IP6K1	Inositol Hexakisphosphate Kinase 1	−0.0174
CACNA2D4	Calcium Voltage-Gated Channel Auxiliary Subunit Alpha2delta 4	0.056197
GPD2	Glycerol-3-Phosphate Dehydrogenase 2	0.001588
MTAP	Methylthioadenosine Phosphorylase	0.019387
GART	Phosphoribosylglycinamide Formyltransferase, Phosphoribosylglycinamide Synthetase, Phosphoribosylaminoimidazole Synthetase	0.005862
DPYD	Dihydropyrimidine Dehydrogenase	0.006427
OAS2	2′–5′-Oligoadenylate Synthetase 2	0.006223
SLC25A27	Solute Carrier Family 25 Member 27	−0.01972
SLC2A8	Solute Carrier Family 2 Member 8	−0.01262
STS	Steroid Sulfatase	0.006514

### Independence Validation of the Prognostic Model

**FIGURE 5:**
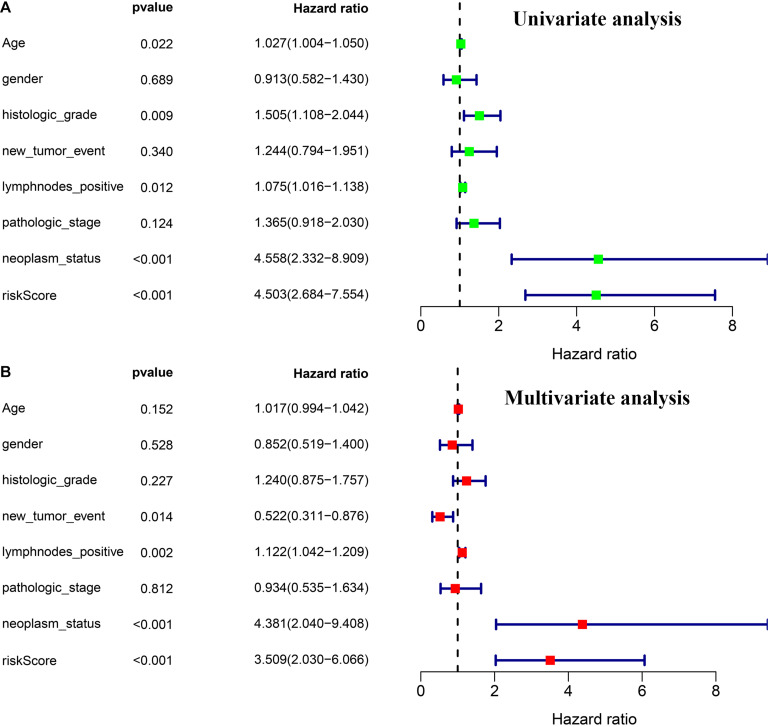
Independence validation of the prognostic model and other clinical features. **(A)** Univariate Cox regression analysis. **(B)** Multivariate Cox regression analysis.

Through univariate and multivariate Cox regression analyses, we found three independent prognostic factors, including risk score, lymph nodes metastasis, and individual neoplasm status (Figures 5A,B).

### Analysis of the Association Between the Risk Score and the Clinical Characteristics

**FIGURE 6:**
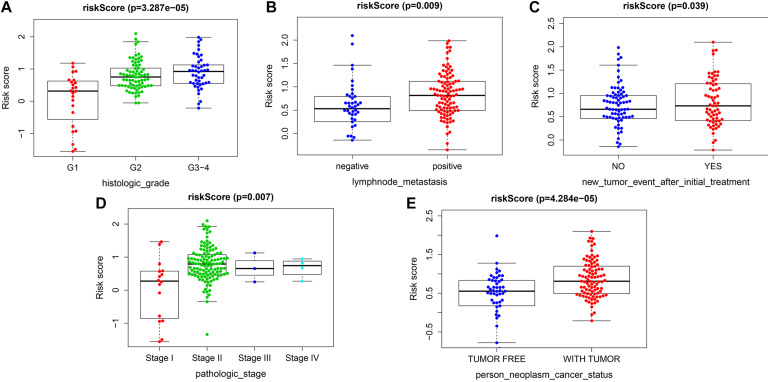
Correlation analysis between risk score and clinicopathological characteristics. **(A)** Histopathological grade. **(B)** Lymph node metastasis. **(C)** New tumor event after initiate treatment. **(D)** Pathological stage. **(E)** Individual neoplasm status.

The risk score exhibited an obvious association with histological grade, lymph node metastasis, new tumor after initial treatment, pathologic stage, and neoplasm status (Figures 6A–E). We also performed chi-square test on the TCGA cohort for analysis of clinical features with different risk groups, the results showed that there were significant differences in lymph node metastasis (*p* = 0.002), personal tumor status (*p* = 0.002), and survival status (*p* < 0.001) among the different risk groups (Table 2).

**TABLE 2:** The chi-square test of the relation between risk score and clinical features in TCGA.

Clinical feature	Risk Score		c2	*p*-Value
	High risk n (%)	Low risk n (%)		
Age			0.155	0.694
>65	37 (48.05%)	30 (44.78%)		
≤65	40 (51.95%)	37 (55.22%)		
Gender			0.218	0.64
Male	43 (55.84%)	40 (59.70%)		
Female	34 (44.16%)	27 (40.30%)		
Histologic grade			2.489	0.115
G1–2	48 (62.34%)	50 (74.63%)		
G3–4	29 (37.66%)	17 (25.37%)		
New tumor event after initiate treatment			0.068	0.794
YES	35 (45.45%)	29 (43.28%)		
NO	42 (54.55%)	38 (56.72%)		
Lymphnodes metastasis			9.666	0.002
Positive	63 (81.82%)	39 (58.21%)		
Negative	14 (18.18%)	28 (41.79%)		
Pathlogic stage			0.346	0.556
I–II	73 (94.81%)	64 (95.52%)		
III–IV	4 (5.19%)	3 (4.48%)		
Person neoplasm status			9.399	0.002
Tumor free	59 (76.62%)	35 (52.24%)		
With tumor	18 (23.38%)	32 (47.76%)		
Survival status			10.833	<0.001
Alive	26 (33.77%)	41 (61.19%)		
Dead	51 (66.23%)	26 (38.81%)		

### External Validation of the Prognostic Model Combined International Cancer Genome Consortium and Gene Expression Omnibus Database

**FIGURE 7:**
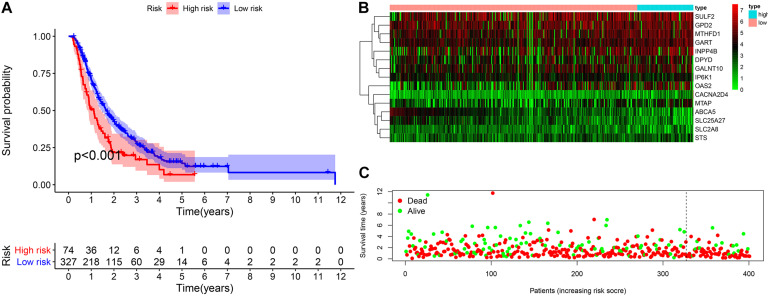
External validation of the prognostic model. **(A)** The Kaplan–Meier curve of overall survival. **(B,C)** The heatmap of the 15 genes and the survival status of patients.

The validation cohort included 401 pancreatic cancer patients from ICGC (PACA-AU and PACA-CA) and GEO databases (GSE62452 and GSE57495). Based on the uniform cutoff value obtained in the TCGA cohort, the group with a high risk included 74 patients, and the group with a low risk included 327 patients. KM survival curve showed that the group with a high risk had a significantly lower OS compared with the group with a low risk (*p* < 0.001) (Figures 7A–C). The AUC values for the risk score predicting OS at 1, 3, and 5 years were 0.589, 0.560, and 0.586, respectively (Supplementary Material 2).

### Gene Set Enrichment Analysis Between Different Risk Groups

**FIGURE 8:**
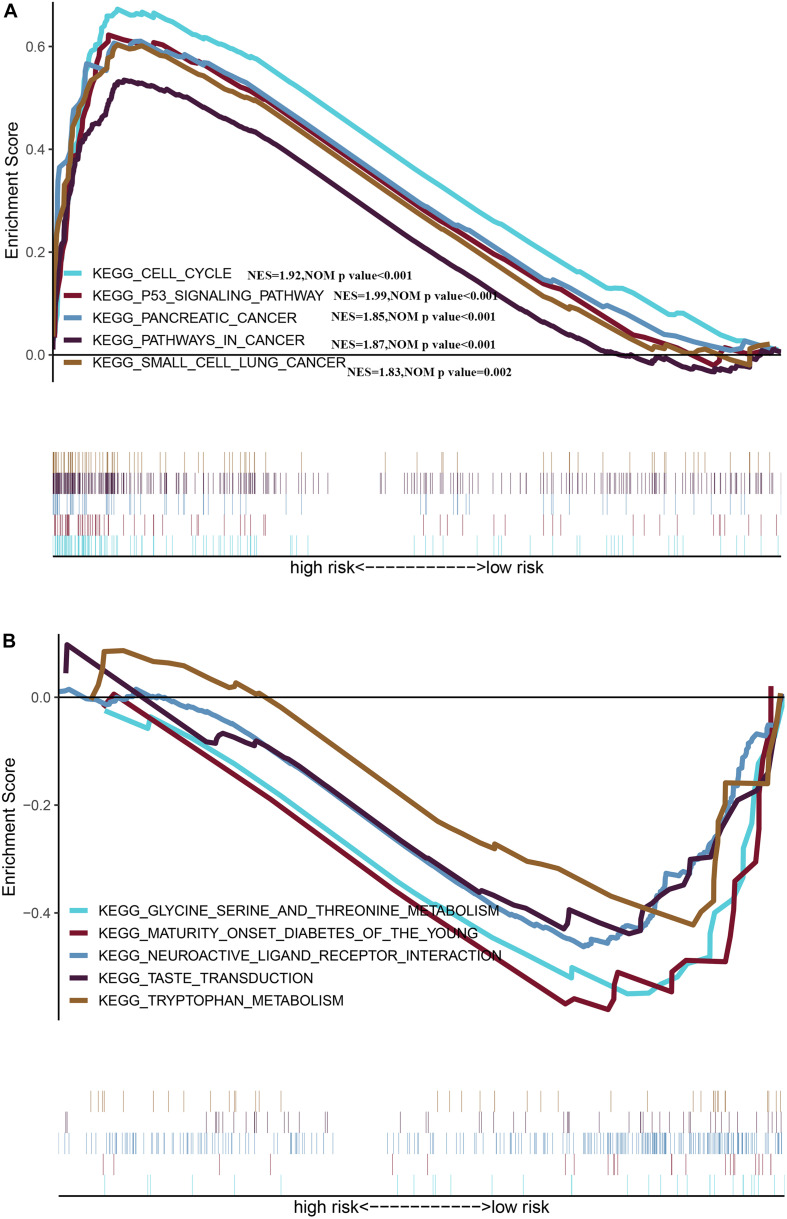
Gene Set Enrichment Analysis between different risk groups **(A)** Multiple GSEA plot of the KEGG pathways enriched for the high-risk group. **(B)** Multiple GSEA plot of the KEGG pathways enriched for the low-risk group.

We identified five oncogenic gene sets with significant enrichment in the group with a high risk: p53 signaling pathway (NES = 1.99, NOM *p*-value < 0.001), pathways in cancer (NES = 1.87, NOM *p*-value < 0.001), cell cycle (NES = 1.92, NOM *p*-value < 0.001), pancreatic cancer (NES = 1.85, NOM *p*-value < 0.001), and small cell lung cancer (NES = 1.83, NOM *p*-value = 0.002) (Figure 8A), while the enriched gene set in the low-risk group was significantly related to metabolism (Figure 8B), indicating that the metabolic activity of the high-risk group was significantly different from that of the low-risk group.

### Tumor-Infiltrating Immune Cells Between Different Risk Groups

**FIGURE 9:**
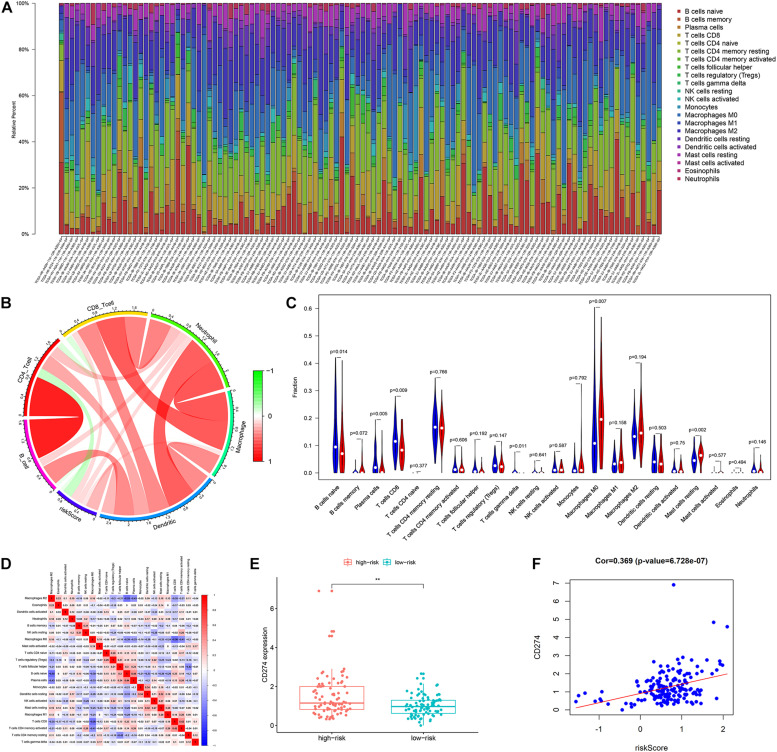
The landscape of immune infiltration in high- and low-risk HCC patients. **(A)** The bar plot of immune infiltration in high- and low-risk HCC patients **(B)** Correlation of the risk score with the immune infiltration of six kinds of immune cells (TIMER method). **(C)** Violin plots visualizing significantly different immune cells between high-risk and low-risk patients (CIBERSORT method, red represents the high-risk group, blue represents the low-risk group). **(D)** Correlation matrix of all 22 immune cells proportions. **(E)** Boxplot plot of the expression level of CD274 between high-risk and low-risk patients. **(F)** Correlation of the risk score with the expression of CD274 (*p*-value significant codes: 0 ≤ ^∗∗∗^ < 0.001 ≤ ^∗∗^ < 0.01 ≤ ^∗^ < 0.05).

The results of the TIMER database showed that there was a negative correlation between risk score and CD4T cell infiltration (Figure 9B). The group with a high risk exhibited an obviously higher infiltration level of macrophage M0 compared with the group with a low risk, while the group with a low risk exhibited an obviously higher infiltration level of B cells and CD8T lymphocytes (Figures 9A,C). There was a negative correlation between macrophage M0, and B cells and CD8T cells (Figure 9D). Besides, the risk score was positively associated with the expression level of CD274 (PDL1) (*r* = 0.369, *p* < 0.001) (Figures 9E,F).

### Building a Survival Predictive Nomogram

**FIGURE 10:**
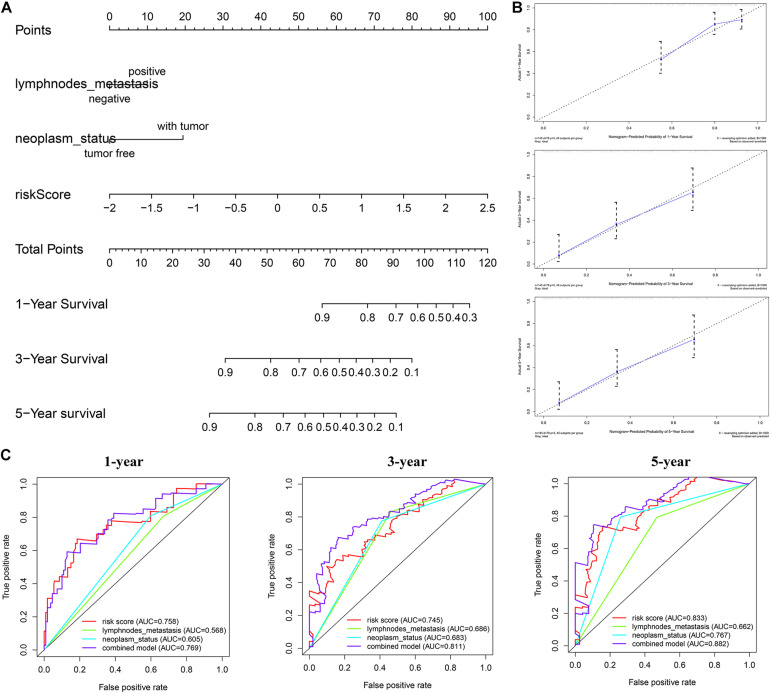
Construction of combined prognostic model in TCGA cohort. **(A)** Nomogram for predicting the probability of 1-, 3-, and 5-year overall survival (OS) for pancreatic cancer patients. **(B)** Calibration plot of the nomogram for predicting the probability of OS at 1, 3, and 5 years. **(C)** Time-dependent ROC curve analyses of the combined prognostic model.

The nomogram we constructed consists of tumor status, lymph node metastasis, and risk score. Each index is an independent factor affecting prognosis. We can estimate patients’ 1-, 3-, and 5-year survival rates based on the cumulative scores of the three indicators (Figure 10A). We used two methods to evaluate the accuracy of the nomogram. The large overlap between the calibration curve and the reference line indicated that the predicted survival rate is close to the actual survival rate, especially in the prediction of patients’ 3- and 5-year survival rate (Figure 10B). The ROC curve demonstrates a better prediction performance exhibited by the combined model compared with a single prediction index (Figure 10C). The concordance index was 0.71, which indicated that the probability of the predicted results consistent with the observed results was high (Supplementary Material 3). Therefore, the combination of risk score and clinical factors can reliably assist in evaluating pancreatic cancer patients’ prognosis.

## Discussion

Pancreatic cancer is a highly malignant digestive tract tumor. Because of its concealed early symptoms, rapid disease progression, low resection rate, and low effective rate of chemotherapy, patients have a very poor prognosis (Ilic and Ilic, 2016). With the accumulation of high-throughput sequencing data, more and more biomarkers have been developed for diagnosing and treating pancreatic cancer. These prognostic signatures involve m6A methylation, autophagy, immunity, and many other aspects (Zheng et al., 2018; Wu et al., 2019; Zhou et al., 2019; Tian et al., 2020; Yue et al., 2020). In recent years, more and more evidence shows that reprogramming metabolism could greatly affect pancreatic cancer in terms of the occurrence, the development, as well as the treatment (Qin et al., 2020). However, the prognostic signatures related to metabolic reprogramming in pancreatic cancer are far from fully cleared.

Patients (572) with complete prognostic information were included in this study. First, we compared 178 pancreatic cancer tissues with 171 normal pancreatic tissues in TCGA and GTEx databases, and identified 379 DEMRGs. Then univariate Cox regression analysis together with the Lasso regression assisted in constructing a novel prognostic model. The unified risk score formula together with the cutoff value were considered to divide patients into a group with a high risk and a group with a low risk. The ROC curve showed the prognostic model with high accuracy in predicting OS, DSS, and PFS of patients. There were 15 genes included into our signature. Among them, ABCA5 is a member of the ATP binding cassette (ABC) transporters, which play a variety of roles in cancer biology and drug resistance. Low expression of ABCA5 is associated with poor prognosis of serous ovarian cancer (Hedditch et al., 2014). Irene Aksoy and others (Aksoy et al., 2017) combined sequencing technology with IPSC technology to identify that GTDC1 is related to neurodevelopmental disorders. Ema et al. (2015) found that SLC25A27 was amplified in advanced gastric cancer with lymph node metastasis. Sulfate endonuclease SULF2 regulates heparan sulfate protein polysaccharide 6-O-sulfation. Alhasan reported that the increase in SULF2 in PDAC is related to advanced tumor stage, vascular invasion, short interval between imaging progression, and short OS (Alhasan et al., 2016). GPD2 is a component of glycerol phosphate shuttle, which can promote the oxidation of glucose, thus, promoting the production of acetyl-CoA. Langston found that GPD2 is involved in the regulation of macrophage inflammation (Langston et al., 2019). MTHFD1 is an enzyme that provides tetrahydrofolic acid-carbon derivatives. Yu found that the high expression of MTHFD1 in hepatocellular carcinoma is associated with a lower survival rate and higher recurrence rate (Yu et al., 2019). Type II inositol polyphosphate 4-phosphatase (INPP4B) is a member of the PI3K/Akt signaling pathway. Zhai found that the overexpression of INPP4B in pancreatic cancer could lead to poor OS and DFS (Zhai et al., 2019). Glycosylation can remarkably affect tumor invasion and immune escape. Zhang found that the high expression of GALNT10 in high-grade ovarian serous cancer (HGSC) is related to immunosuppressive microenvironment, thus promoting tumor progression (Zhang et al., 2020). No reports focus on studying the effect of the remaining genes on cancer.

The group with a high risk presented a worse prognosis compared with the group with a low risk. The external validation results of the ICGC and the GEO cohorts further confirm the effectiveness of this prognostic model. GSEA revealed the oncological characteristics with significant enrichment in the group with a high risk, and pancreatic cancer is one of them, while the group with a low risk was associated with multiple metabolic pathways, indicating that the imbalance of tumor metabolic microenvironment may affect the progression of pancreatic cancer. The tumor microenvironment is a hot topic in the field of tumor research in recent years. Multiple studies have shown that metabolic reprogramming can have a significant impact on the tumor microenvironment (Lyssiotis and Kimmelman, 2017; Reina-Campos et al., 2017). Immune cells are an important component of the tumor microenvironment, which has been proved to be valuable in predicting the prognosis of tumors (Gentles et al., 2015). YIno found that tumor-infiltrating CD8T cells can be used to independently predict the prognosis of pancreatic cancer, and the high infiltration of CD8T cells is associated with longer survival (Ino et al., 2013). In this study, we also found that the proportion of CD8T cell infiltration in the group with a low risk was higher than the group with a high risk, further confirming the prognostic value owned by tumor-infiltrating CD8T cells in pancreatic cancer. Programmed cell death ligand 1 is one protein encoded by the CD274 gene. When it binds to PD1, it transmits a negative regulatory signal to T-cells, induces T-cells to enter a resting state, reduces the proliferation of CD8T cells in lymph nodes, making them unable to recognize cancer cells, reduces T-cell proliferation or apoptosis, effectively relieves the immune response of the body, and promotes further proliferation of cancer cells (Chen and Han, 2015; Naidoo et al., 2015). This study found that the risk score was positively related to the expression level of CD274 (PDL1), so the group with a high risk exhibited a poor prognosis possibly caused by the mechanism of immune escape. Besides, we can also predict the degree of tumor differentiation, clinicopathological stage, and lymph node metastasis according to the risk score, which has important reference value for clinical decision making. As revealed by the univariate and multivariate Cox regression analyses, individual neoplasm status, lymph node metastasis, as well as risk score were independent predictors of prognosis. We combined three indicators to construct one nomogram for the prediction of 1-, 3-, and 5-year OS of pancreatic cancer. The nomogram further enriches the prognosis evaluation system of pancreatic cancer, and the predictive ability of the risk score is further improved. The nomogram has a better prediction effect than a single predictor.

The study integrated as well as analyzed high-throughput sequencing data from multiple databases, and a personalized nomogram for survival prediction was gradually created. However, due to the lack of corresponding clinical data in the validation queue, we only performed internal validation on nomogram. Metabolic genes in the model may be potential targets for diagnosis or treatment of pancreatic cancer, and their detailed mechanisms need to be explored with the help of *in vivo* and *in vitro* verification experiments. This study is only a retrospective study, and further prospective results are needed to support each other.

## Conclusion

The study focused on constructing a signature and a nomogram associated with metabolic reprogramming for predicting the prognosis of pancreatic cancer, which may help to further improve the treatment strategy of pancreatic cancer.

## Data Availability Statement

The original contributions presented in the study are included in the article/Supplementary Material, further inquiries can be directed to the corresponding author/s.

## Author Contributions

JH and LW designed this study. JH analyzed the data in this study, interpreted the findings, and drafted the manuscript. LW and YZ carried out data management and revised the manuscript. All authors reviewed the final version of the manuscript.

## Conflict of Interest

The authors declare that the research was conducted in the absence of any commercial or financial relationships that could be construed as a potential conflict of interest.

## Footnotes

1 https://portal.gdc.cancer.gov/

2 https://xenabrowser.net/

3 https://icgc.org/

4 https://www.ncbi.nlm.nih.gov/geo/

## Supplementary Material

Supplementary Material 1 The risk score distribution of TCGA.

Supplementary Material 2 The time-dependent ROC curve for external validation.

Supplementary Material 3 Concordance index for the nomogram.

## Abbreviations

TCGA: The Cancer Genome Atlas
ICGC: International Cancer Genome Consortium
GEO: Gene Expression Ominibus
GTEx: genotype-tissue expression comprehensive database
DEMRGs: differentially expressed metabolic-related genes
GO: Gene Ontology
KEGG: Kyoto Encyclopedia of Genes and Genomes
GSEA: gene set enrichment analysis
RS: risk score
HR: high risk
LR: low risk
KM: Kaplan–Meier
ROC: receiver operating characteristic
AUC: the area under the curve
OS: overall survival
DSS: disease special survival
DFS: disease-free survival
PFS: progression-free survival
FDR: false discovery rate.
