# Supplementary figures and images for: Development and Validation of a Novel Metabolic-Related Signature Predicting Overall Survival for Pancreatic Cancer
Source: Front Genet. 2021 May 28;12:561254. doi: 10.3389/fgene.2021.561254 (PMC8194314; doi:10.3389/fgene.2021.561254)

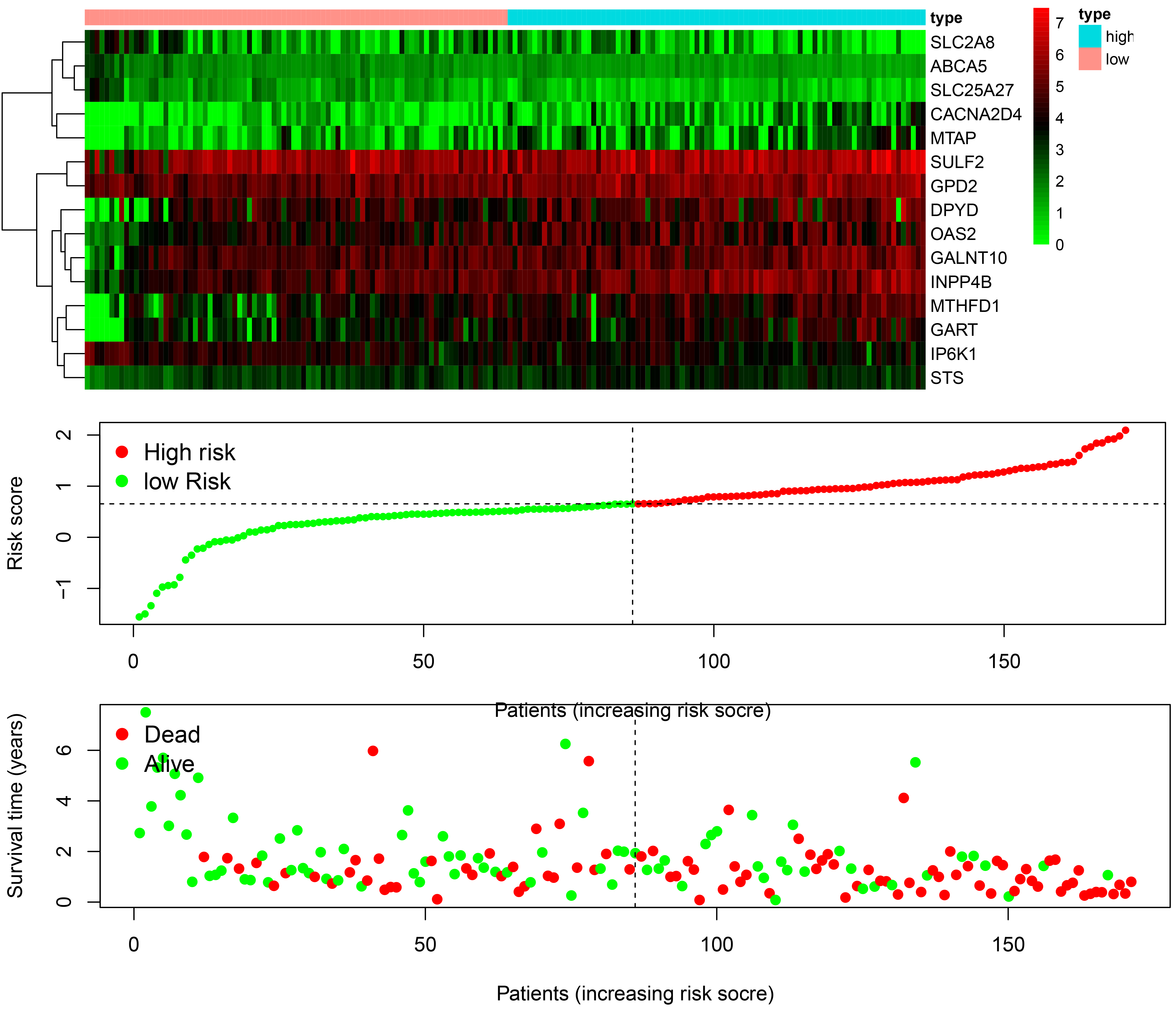

Supplement: Supplementary Material 1 — The risk score distribution of TCGA. [file Image_1.TIF]

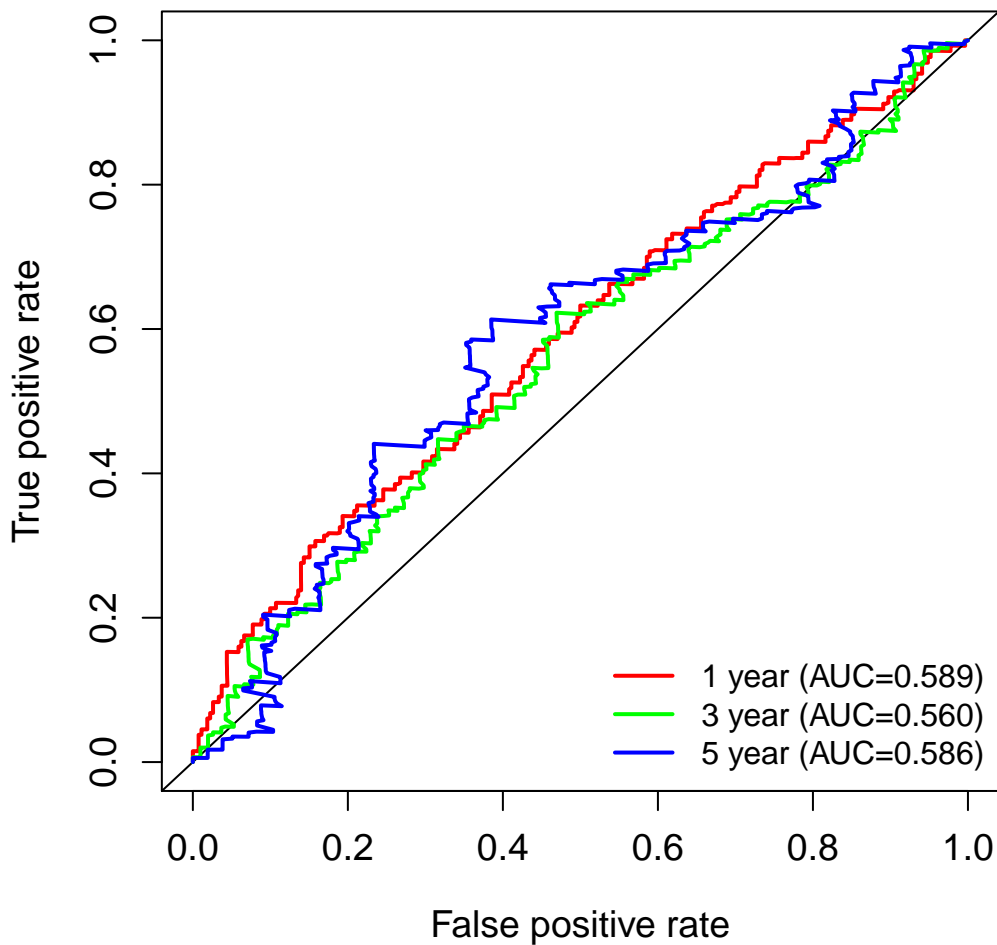

Supplement: Supplementary Material 2 — The time-dependent ROC curve for external validation. [file Image_2.PDF]

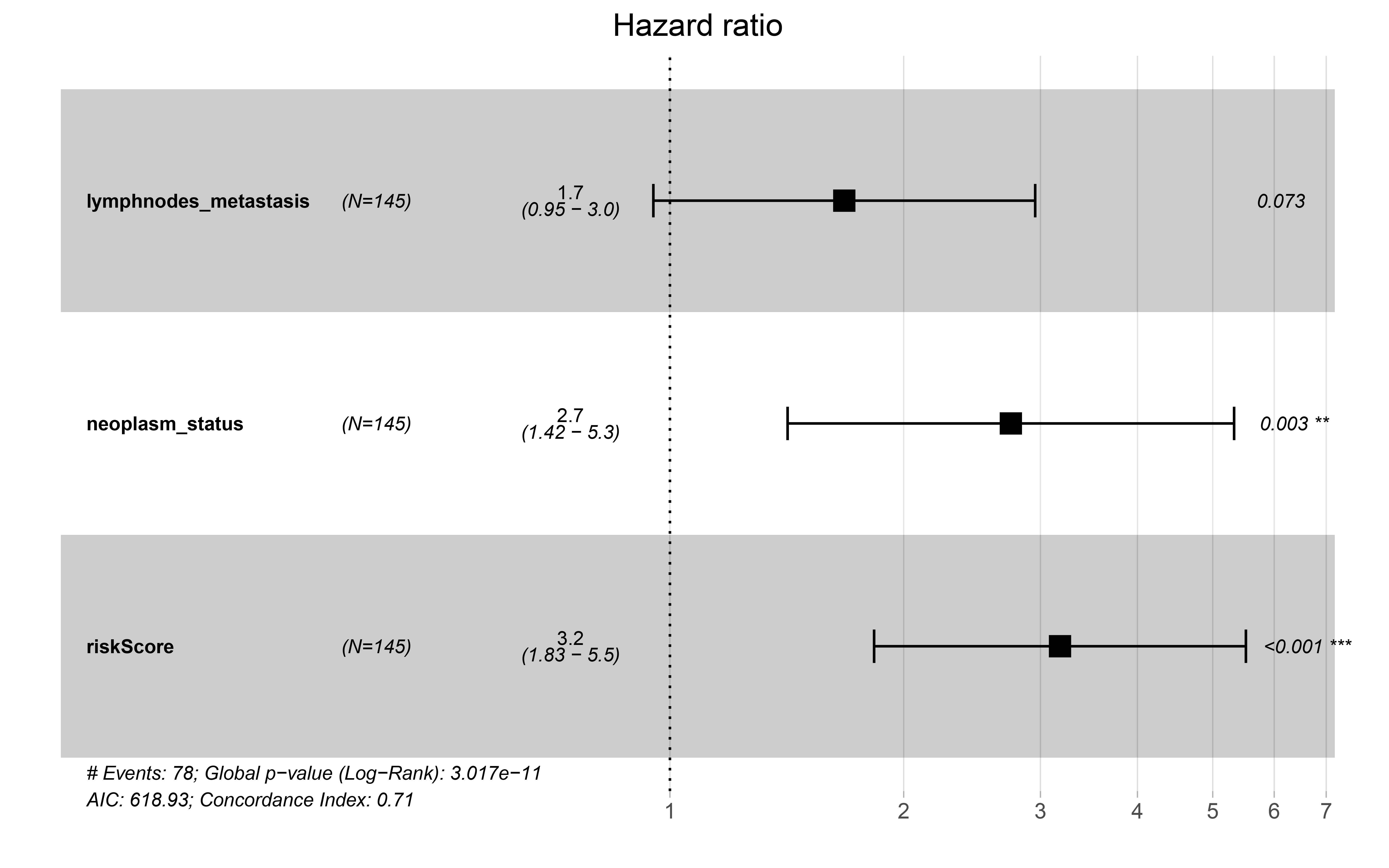

Supplement: Supplementary Material 3 — Concordance index for the nomogram. [file Image_3.TIF]
